# The Vitality of Public Space and the Effects of Environmental Factors in Chinese Suburban Rural Communities Based on Tourists and Residents

**DOI:** 10.3390/ijerph20010263

**Published:** 2022-12-24

**Authors:** Jie Zheng, Junjun He, Hongya Tang

**Affiliations:** 1 School of Textile Engineering and Art, Anhui Agricultural University, Hefei 230036, China; 2 School of Geography and Tourism, Anhui Normal University, Wuhu 241003, China

**Keywords:** suburban rural community, public space, space vitality, environmental factors

## Abstract

The vitality of public space in rural communities is an important symbol of rural revitalization, especially in suburban rural communities. Previous studies focused on rural industries and ignored the effects of the spatial environment on different groups of people. Hence, this study takes the public space of suburban rural communities as an example and uses Global Positioning System (GPS) and cognitive mapping data to establish a new vibrancy assessment system for tourists and residents, respectively. The effects of the public space environment and space vitality in suburban rural communities are revealed through ordinary least squares (OLS) and geographically weighted regression (GWR) models. The results suggested that: (I) There were pronounced seasonal changes and spatial distribution differences in the space vitality of tourists, while residents were concentrated in fixed public spaces. (II) For tourists, the public space vitality in rural communities was affected by seven factors, including accessibility, seats, green looking ratio, recreational facilities, water area, plant species richness, and plant color composition. Green looking ratio and water area had a negative impact. For residents, the public space vitality in rural communities was affected by five factors, including shelter facilities, seats, accessibility, space type, fitness facilities. Only fitness facilities had a negative effect. Our research proposed a feasible and effective method to assess the vitality of rural public space in rural communities, and the finding from this study provides significant implications for the development and planning of suburban rural communities oriented by vitality.

## 1. Introduction

Rural recession is a challenge for many countries [1]. In the face of this issue, China has been trying to solve it through urban-rural transformation. During China’s rural revitalization over the past decades, the spatial structure and function of rural communities have changed dramatically. The development of rural communities has shifted individual functions to diversified functions [2]. The report of the 20th National Congress of the Communist Party of China held in October 2022 emphasized the integrated development of rural communities once again, proposing to “promote rural revitalization comprehensively, and giving priority to the development of agriculture and rural areas”. Improving the living environment and enhancing the vitality of rural communities are important indicators of rural revitalization. Meanwhile, this brings new challenges to the sustainable development of rural communities. China’s urbanization is characterized by urban-rural integration. Against this background, suburban rural communities are particularly important since they are bridges between urban and rural areas. Therefore, the way to take a differentiated approach to enhance the vitality of suburban rural communities is of vital practical significance to direct sustainable development.

Suburban rural communities grow faster than general rural communities far from urban areas in terms of urbanization and agricultural modernization, which is attributable to their unique strengths in location, transportation, economy, landscape environment, etc. Accordingly, they have turned into a frontier and key area of rural revitalization, and their development model and spatial structure determine the process of urbanization [2]. Furthermore, suburban rural areas are affected by both their development and the spillover of urban functions. The migration changes of the rural population in the suburbs have changed the original closed nature of rural areas, and more and more heterogeneous social relations have rushed into rural areas, which affect the culture, life and industries in rural areas to varying degrees [3]. In addition, since high-density urban spaces can no longer meet people’s needs for leisure, people are increasingly consuming the rural areas in search of nostalgia, and suburban rural communities close to cities naturally become the first choice for urban people. While the growth of tourism has laid the economic foundation for many rural communities, it has also had several negative impacts. These impacts are not only on the natural and cultural environments but especially on residents and tourists in rural communities [4,5]. To alleviate such negative effects, many scholars have proposed that the needs and behaviors of various groups in society should be considered [6,7,8]. As the spaces of experience for tourists, the windows into the communal life of residents, and the important resources and assets of rural collectives, public spaces in rural communities are of great significance to the development of rural communities. Theoretically, rural public space is the core of community design and plays an important role in the relationship between various groups of people [9,10]. Hence, community public spaces need to be consciously established to address the environmental needs of various groups of people to enhance space vitality.

In order to address the aforementioned needs, this study introduces “space vitality” to measure the public space, which is constituted by group behaviors and the spatial environment [11]. Current studies on space vitality mostly focus on urban space [12,13]. There are also some studies on rural communities in general [14,15]. However, studies of suburban rural communities focus more on industry and tourism [16,17] and less on the vitality of the suburban rural public space itself. Space is a carrier of people and their behaviors, which are affected by the physical difference and environment of space. Tourists and residents are key sources of public space vitality in suburban rural communities. Since they have different positions and conclusions [18,19], rural public space planning should address the needs of both hosts and guests [20]. For instance, the need for key space planning (the activity space for residents, the integrated space for residents and tourists, and the experiential space for tourists) was proposed based on the renewal of Gu-Ryong Village in South Korea according to Kim and Choi’s proposal [21]. The preference of residents and tourists for public space landscape is a strong stimulator for the formation of the vitality of rural communities [19]. The ability of groups to use public spaces and the public space vitality are artificially controlled through arrangements suitable for the space environment [22,23].

However, the variation and development of public spaces in rural communities are dynamic. It has been a challenge to determine the vitality of rural public space. The study approach for assessing the vitality of rural space included principally traditional field observation and questionnaires. However, such approaches require a lot of time and manpower. Moreover, questionnaire data may be biased by the subjectivity of respondents. With the rapid development of the internet and big data, the spatiotemporal behaviors of groups, the state of rural operations, etc., can be monitored in real-time. For instance, cellular signaling data [24], Baidu heat map [25], GPS [26] and night light data [27] could effectively and efficiently record spatial locations, time, etc., of rural group activities, thereby offering new vision and technical support for current studies on the vitality of suburban rural communities. On the other hand, figuring out what factors affect the vitality of public space in suburban rural communities is an important issue for sustainable rural development. At present, many studies have found that both physical attributes (such as spatial scale, accessibility, and spatial quality) are associated with space vitality [28,29], although describing space vitality around “human activities” and exploring the influencing factors of space vitality has become the main research path for studying space vitality [30,31]. While describing space vitality around “human activities” and exploring the factors influencing space vitality have become the main research paths in studying space vitality. However, few studies focus on public spaces in suburban rural communities and explore the relationship between different groups of people and spatial environments.

Therefore, the relationship between the vitality of public space and the environment in suburban rural communities was explored from the perspectives of residents and tourists in the study. With Shecun Community, Jiangning District, Nanjing as an example, the study evaluated the public space vitality in suburban rural communities from the perspective of residents and tourists, probed into the relationship between public space vitality and environmental impact factors through the ordinary least squares (OLS) and geographically weighted regressions (GWR), and discussed the vitality optimization strategy for public spaces in suburban rural communities on this basis. The study is focused on the following: (1) Spatio-temporal differentiation characteristics of tourists and residents in public spaces of suburban rural communities; (2) The effects of various environmental factors of public spaces on activities of various groups in suburban rural communities; and (3) How to build public spaces that meet the needs of residents and tourists.

## 2. Materials and Methods

### 2.1. Study Area

**Figure 1:**
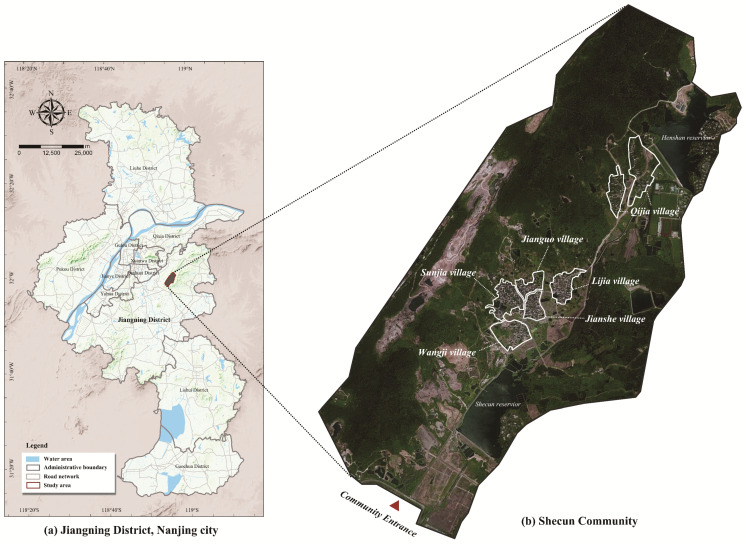
Location map of Shecun Community, Jiangning District, Nanjing, Jiangsu Province (**a**). Geographic Location of the Study Area (**b**) Shecun Community.

The subject of the study is Shecun Community, Jiangning District, Nanjing, Jiangsu Province (Figure 1). This rural community was chosen as the case study area for three reasons. First, located in Jiangning District, Nanjing, Shecun Community is a suburban rural area of Nanjing. It is approx. 13 km southeast of the seat of the district government, boasting convenient transportation. With an urbanization rate of 83.2%, Nanjing is not only the capital of Jiangsu Province but also one of the most developed international cities in China. Second, Shecun Community, as one of the first pilot villages with the most abundant in Jiangsu Province, is one of the villages with the most typical “landscape and pastoral” settlement in Jiangning and is one of the villages with the richest traditional elements of “history and humanities” in Jiangning. Situated in the Mt. Qinglong—Mt. Dalian Ecological Corridor, this community connects with the Shecun Reservoir in the south, meets with Mt. Qinglong in the west and joins Mt. Heng in the north, featuring an ecological location surrounded by mountains on three sides and water on one. The total land area of the Shecun Community is 1577.08 hectares, and the area of land for construction is about 48.51 hectares, accounting for 3.08% of the total land area. The area of non-construction land is 1358.47 hectares, accounting for 86.14% of the total land area. Agricultural and forestry land account for a dominant proportion at present. There is a certain amount of state-owned land for construction on both sides of the reservoir. In the southwest are mostly mining land that needs to be remediated, and the remaining mine pits are tourist attractions of the Shecun Community. Since its construction, it has developed into an increasingly popular tourist destination in the suburbs. This community received over 100,000 tourist-times in 2019 and 150,000 in 2020, and the income from tourism amounted to RMB 20 million. Third, the relationship between residents and tourists is obvious in Shecun Community because the activity space of residents is very close to that of tourists. With 2585 permanent residents, the Shecun Community consists of six natural villages, i.e., Sunjia Village, Wangjia Village, Jianshe Village, Jianguo Village, Lijia Village, and Qijia Village. Wangjia Village is a characteristic pastoral village. Sunjia Village is a model village, Jianshe Village. Jianguo Village and Lijia Village are key remediation villages, and Qijia Village is a general village. The rural community is not only the place of residence of rural residents but also the best choice for urban residents to travel short distances. The study subjects identified in this paper can not only provide space for leisure, entertainment, culture and other tourism activities for urban residents but are also communities that retain a certain local rural pattern and are inhabited by lots of native residents. Hence, the Shecun Community, as a typical suburban rural community, is worth studying.

**Figure 2:**
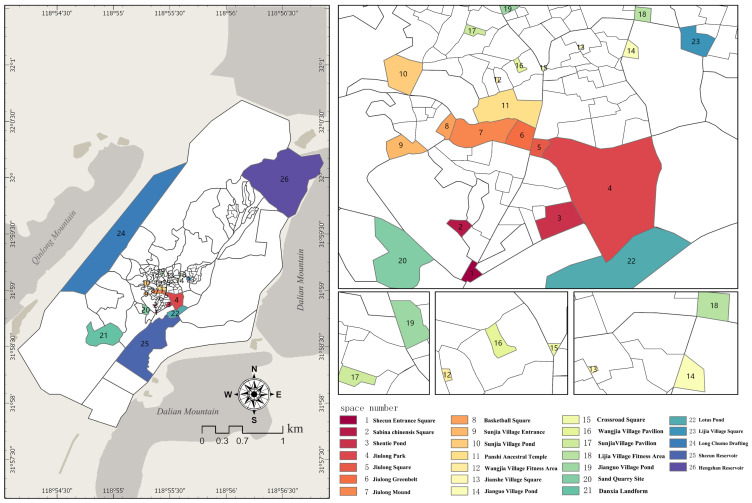
The layout of the Shecun Community and Public Space.

Since community public space is principally designed to provide users with spaces for various activities such as socializing, playing, walking and dancing, it is essential to consider the number and duration of users, as well as the landscape configuration when choosing such spaces. Hence, a preliminary field investigation of the study area was conducted concerning the plan of Shecun. Then the sample area was chosen based on the tourist attractions and usage by residents and tourists to exclude completely unused spaces. Accessible public spaces were chosen in the typical sampling principle of Braun-Blanquet School. The scope of the sample area was determined by taking forest edges or road edges as boundaries whenever possible. Large-area sites with unclear boundaries were determined based on their actual use, and 26 community spaces were finally identified in the principal area above (Figure 2).

### 2.2. Study Data

#### 2.2.1. Environmental Characteristics

First, the study identified the environmental factors of field investigation, including space form, plant landscape, topography and geomorphology and related facilities, concerning the existing literature [32,33]. Refer to Table 1 for details. Then, the site survey data, the orthographic projection images, and the front projection map (DJI Drone Mapping) images were taken as data sources of environment and space information. Finally, software tools ArcGIS Pro and CAD were used to extract the variable values of various spatial characteristics.

#### 2.2.2. Space Vitality

With the advent of the era of big data, research data sources have expanded to network channels, such as using Baidu heat maps and POI data to quantify urban space vitality [32]. However, these data are more suitable for regional studies, and the village scale needs to explore population activities from a more micro-scale. The data for space vitality is divided into two parts, residents and tourists. Field investigation showed that activity areas and behavior patterns of residents were quite different from that of tourists. Tourists had a constant demand for sightseeing, and they were not familiar with tourist attractions. Therefore, their demand for places can be accurately depicted by determining complete tour tracks with a hand-held GPS. Residents mostly stay in a fixed space regardless of the season and time. Hand-held GPS was of little significance to the identification of residents’ short-term activities. Hence, they were investigated by a cognitive map questionnaire. The detailed description is as follows.

**Figure A1:**
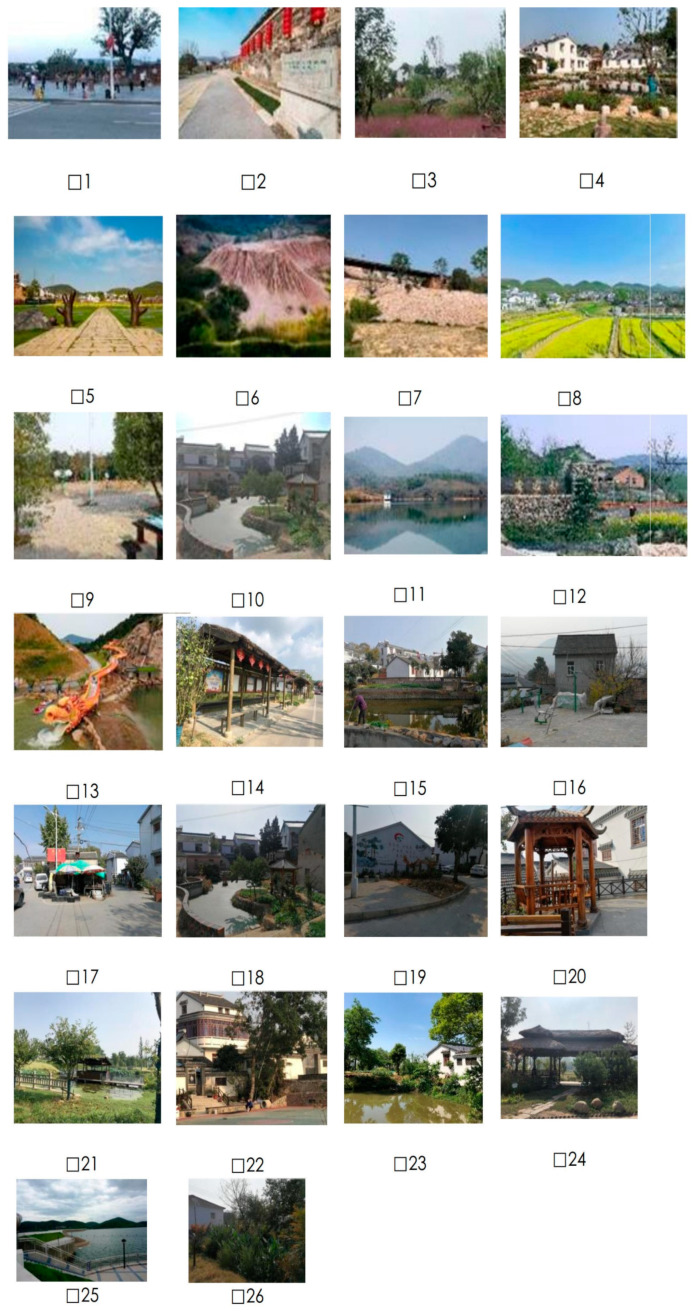
Resident Cognitive Questionnaires.

For the residents, the research team, assisted by the Party-Public Service Center of the Shecun Community, handed out a total of 1400 anonymous questionnaires to residents from September 2019 to December 2019. The cognitive questionnaire contained photos of all 26 public spaces in the Shecun Community (Figure A1). A total of 1400 copies were recovered, and 1384 were returned, with an effective rate of 98.8%. They corresponded to the space with the ArcGIS Pro software to make statistics of the public spaces in rural communities that were known by residents, thereby roughly determining the scope of daily activities of the permanent residents in Shecun. The concept of the cognitive map originated from American psychologist Tolman [33], and it is intended to capture people’s intrinsic cognition of and preference for the environment. It can investigate and grasp simple and specific affairs to make the investigation simple and easy.

The study surveyed the tourists three times a month (including holidays, weekdays and weekends) between June 2018 and June 2019. The spatiotemporal behavior data of tourists with personal attributes were acquired utilizing hand-held GPS and the mating questionnaire survey. The portability, diversity and availability of GPS positioning and tracking, as a new investigation method for studying human behavior, have been extensively recognized in the research field [34,35]. Since GPS could only acquire data on behavior trajectory, the participants needed to fill out the mating questionnaire when returning the GPS. On the one hand, this gave full play to the advantages of GPS in positioning accuracy. On the other, the questionnaire survey could work with a personal spatiotemporal trajectory to reveal personal attributes, which is convenient for data collation and a more accurate grasp of tourists’ behaviors. The survey comprises three key steps, i.e., device distribution and explanation, survey process, and device recovery. Since most tourists in suburban rural areas take public transport or use private cars, Shecun’s bus stops and parking lots for private cars were chosen as locations of the survey so as to reduce redundant records. A total of 234 GPS devices were distributed, and 221 effective trajectories were collected, with an effective rate of 94.4%. Explorer V900 hand-held GPS devices with a time accuracy of 1 second (positioning every second) and a receiving sensitivity superior to −165 dBm was used in the survey. Their positioning accuracy was as high as 2.5 m/CEP (50%) and 8.0 m/CEP (95%) without DGPS differential correction and up to 1.5 m/CEP (30–50%) and 2.5 m/CEP (95%) with DGPS differential correction, which ensured accurate identification of tourists’ time locations.

### 2.3. Analysis Method

**Figure 3:**
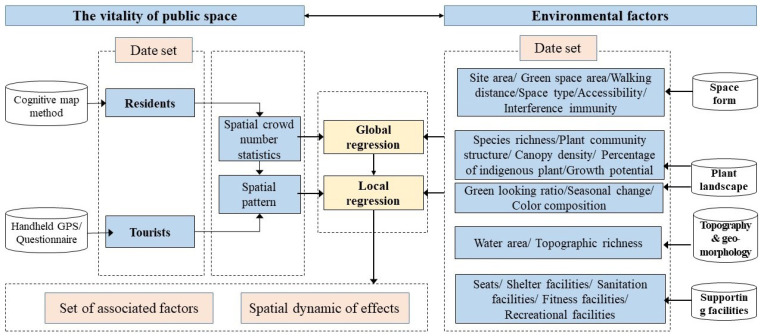
Analysis Framework.

The study was divided into three parts. Firstly, the vitality of residents and tourists in 26 public spaces was quantified from the cognitive map questionnaire and GPS trajectory data. Spatio-temporal differentiation characteristics were analyzed on this basis. Then, the environmental indicators for public space in a suburban rural community were established using four dimensions: space form, plant landscape, topography and geomorphology, and supporting facilities. Next, the OLS model and Moran’s I Index were used to evaluate and screen appropriate explanatory variables in environmental factors. Finally, the GWR model probed into the relationship between the environment and the vitality of tourists and residents in public spaces, as shown in Figure 3.

#### 2.3.1. Quantified Space Vitality

The characteristics of crowd activities directly reflect the vitality of the space. Therefore, in this study, the number of users gathered in a specific range of space was used to characterize space vitality. The users of public space are divided into tourists and residents in suburban rural communities.

For the residents, the resident behavior data acquired with the cognitive map was imported into the ArcGIS Pro software as shapefile data with strict reference to its number and location, a series of point data was yielded, and the information about each resident in the behavior record form was correlated to the point data on a one-to-one basis by linking with the attribute table, so as to visually express the residents’ behavior data. Then, the counting feature in the attribute table of ArcGIS Pro software was used to count the number of residents in each public space. For the tourist, Everphoto V1.9.5.3 (the mating software for the Explorer GPS) was used to filter out and delete the trajectory data of tourists involving incomplete trajectory data, except in time interval data, and GPS data in abnormal data format, thereby yielding effective data. The processed data was then imported into ArcGIS Pro. Finally, hierarchical classification of the number of residents and tourists was performed for an intuitive presentation of the public space vitality in rural communities.

#### 2.3.2. Screening of Environmental Factors

In the study, OLS models were used to screen for reasonable explanatory variables (environmental factors). However, before conducting the OLS model analysis, spatial correlation tests of the study variables are required to assess the presence of spatial heterogeneity. The study mainly used Moran’s index (Moran’s I) to test the spatial autocorrelation of regional variables [36]. The computing formula is as follows:

$$I=\frac{n}{\sum_{i=1}^{n}\sum_{j}^{n}W_{ij}}\times \frac{\sum_{i=1}^{n}\sum_{j}^{n}W_{ij}(x_{i}-\bar{x})(x_{j}-\bar{x})}{\sum_{i=1}^{n}{\left(x_{i}-\bar{x}\right)}^{2}}$$

where $x^{i}$ and $x^{j}$ are values of the chosen variables, $W^{ij}$ is the geospatial weight connection matrix of *i* and *j*, and *x* denotes the average population of all community units. The Moran’s I statistic ranges from [−1, 1], with I > 0 indicating a positive correlation. When I = 0, it is irrelevant. When I < 0, it indicates a negative correlation.

To ensure the accuracy and rationality of the GWR model, the alternative independent variables of the model need to satisfy certain conditions, namely, the absence of severe multi-collinearity in the alternative independent variables and the presence of instability between the explanatory and dependent variables [37]. Therefore, the alternative independent variables need to be analyzed and checked from these two aspects before GWR analysis. The variance inflation factor (VIF) in OLS was used for multi-collinearity detection of explanatory variables in the study. Multi-collinearity means there is a highly linear relationship between independent variables in the linear regression model, to avoid significant deviation of an estimated result of the model from the real value and to guarantee the accuracy and rationality of the model result. As shown in the computing formula:

$$VIF=\frac{1}{\left(1-r^{2}\right)}$$

where *r* denotes the determination coefficient of linear regression, representing the percent change of the explanatory dependent variable for the linear regression equation. The greater the VIF, the higher the possibility of collinearity between explanatory variables. When 0 < VIF ≤ 10, there is no high degree of multi-collinearity between effect variables, variables can be used for regression analysis, while the rest of the explanatory variables should be deleted.

#### 2.3.3. Geographical Weighted Regression Analysis

Through the investigation of stepwise linear regression of each dependent variable and independent variable, the independent variables without strong multi-collinearity and with an effect on dependent variables were chosen for GWR analysis. In the analysis of traditional linear regression models for spatial statistical analysis of geography, it was assumed that regression parameters were independent of the geographical properties of the sample data and that the regression parameters obtained through calculation by the least square method are not only the best-unbiased estimators of sample data but were also the best-unbiased estimators of all sample data throughout the study area. Besides, scholars found that the regression parameters of sample data exhibited significant heterogeneity in different geographical locations in the study of practical problems. Therefore, the parameter estimates yielded with the linear regression model could not accurately reflect the geospatial characteristics of regression parameters. The regression parameters in the GWR model are functions that represent geospatial locations [37] and constitute the extension and improvement of the linear regression model. The spatial weight matrix was used in the linear regression model through the following computing formula:

$$y_{i}=\beta_{0}\left(u_{i},v_{i}\right)+\sum_{k}\beta_{k}\left(u_{i},v_{i}\right)X_{ik}+\varepsilon_{i}\;\;\;i=1,2,\ldots ,n$$

where ($u_{i}$, $v_{i}$) is the coordinates of the *i*-th sample point (e.g., space latitude and longitude), and $\beta_{0}$($u_{i}$, $v_{i}$) is a constant term, $\beta_{0}$($u_{i}$, $v_{i}$) represents the regression coefficient of the *k*-th explanatory variable at the *i*-th sample point and is the function of geospatial location, $X_{ik}$ is the *k*-th explanatory variable of the *i*-th sample point, and $\varepsilon_{i}$ ~ *n*(0, δ2) denotes random error.

## 3. Results

### 3.1. The Spatial Distribution of Space Vitality

#### 3.1.1. Residents

**Figure 4:**
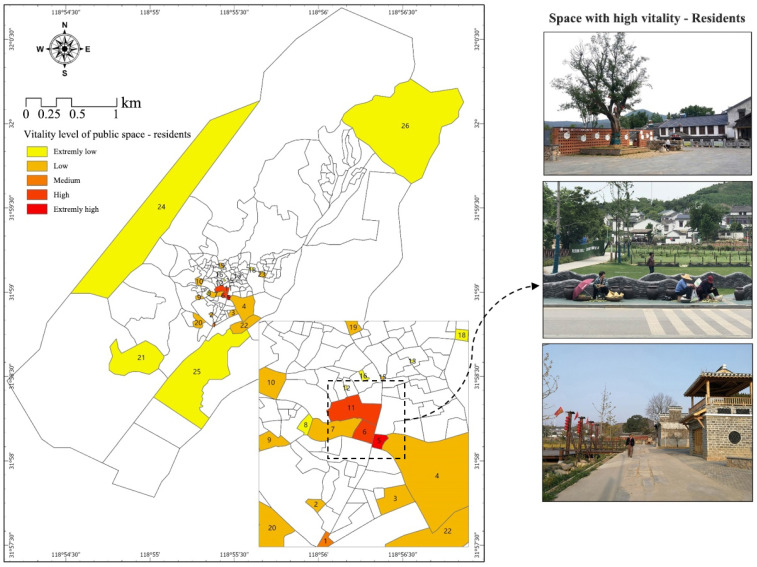
Public Space Vitality—Residents.

The spatial distribution map of residents’ vitality was quantified with reference to the cognitive map (Figure 4). Overall, the vitality distribution of public space presents a spatial pattern of a high-value single core, decreasing to the periphery. As shown in the map, the area with high awareness among residents in Shecun is space 5, and the next areas with high vitality are public spaces 6 and 11. This means residents only carried out activities in these public spaces, resulting in a high level of vitality therein among residents. According to the field survey, it was found that space 5 was a square, which was a space for residents to carry out various gathering activities like square dancing, chatting, and selling behavior. It should be noted that residents of the Shecun Community spontaneously held selling activities, especially on weekends and holidays, when residents sold things in the marginal areas of spaces 5 and 7. For residents, less profitable spaces, especially the public spaces away from central areas and the spaces inside alleys and lines, accounted for a large proportion. The spaces inside alleys and lanes included public spaces 18, 14 and 12. The field investigation showed that several fitness spaces and recreational lookout pavilions were established to improve the healthy quality of life of rural residents during urbanization development, but such spaces were rarely occupied due to their poor spatial accessibility, residents’ habitual cognition, etc.

#### 3.1.2. Tourists

**Figure 5:**
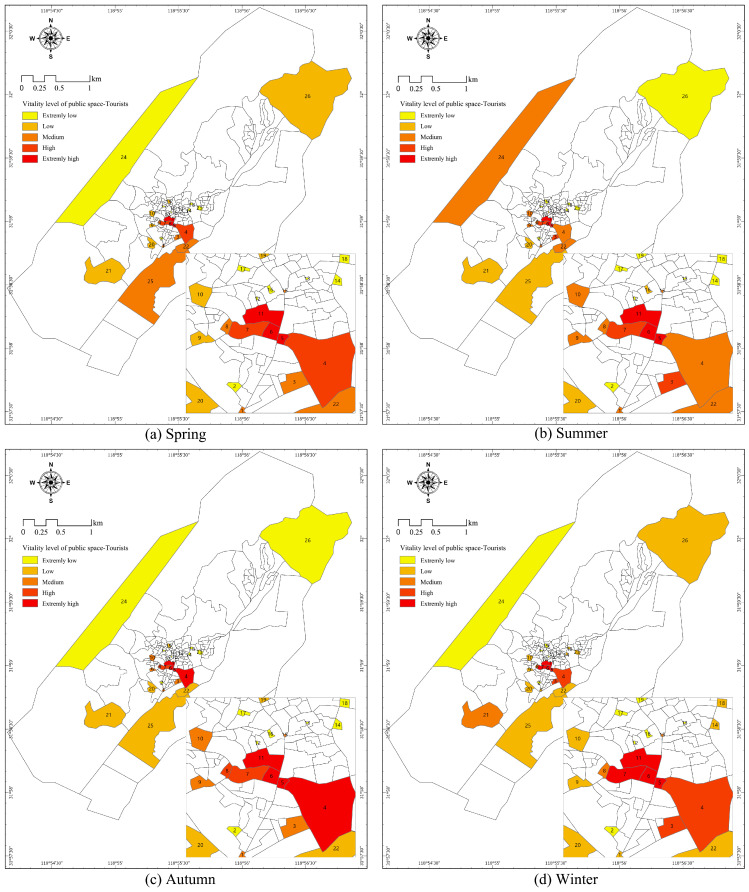
Four Seasons Public Space Vitality—Tourists.

Based on the quantified tourist data, the number of group activities per tourist in the public space of Shecun was classified by the seasons of spring, summer, autumn and winter (Figure 5). Furthermore, the intensity of tourists’ behavioral activities could be divided into five categories, i.e., extremely high vitality, high vitality, medium vitality, low vitality and extremely low vitality, through the classification of natural discontinuities. The spatial distribution of tourists’ vitality in the four seasons was similar: that is, all public spaces with high vitality were located in the central area of the Shecun Community and linked up into vibrant areas. By contrast, the low-vitality public spaces in the four seasons were scattered since they were normally located at the margin of Shecun Community or in the alley and lane spaces.

In spring, the public spaces with extremely high vitality, high vitality and medium vitality in the rural communities linked up into vibrant areas, the public spaces in the central area had the highest vitality, which gradually weakened towards the surroundings. In other words, tourist activities were concentrated in these public spaces, e.g., spaces 5, 6, 7 and 11. In summer, spaces 5, 6 and 11 remained highly vibrant. The vitality of public space 22 and the vitality of public space 24 at the margin of Shecun Community reached their maximum and was high in summer. It was found through field investigation that this is the location of a rafting project called “Here Be Dragons”, which was only open in summer. It can be understood that the vitality of these two public spaces was greatly influenced by tourists, and there were obvious seasonal differences. In autumn, the public spaces of Shecun Community, on the whole, presented a linear and highly concentrated spatial pattern of vitality, where the vitality intensity did not decay progressively from the center to the periphery but exhibited a cliff decline in space. Public spaces with extremely high vitality and high vitality included public spaces 4, 5, 6, 7, 8 and 11, all of which were on an axis. In winter, the spatial distribution characteristics of vitality in public spaces of the Shecun Community were similar to those in spring, with spaces 5, 6, 7 and 11 as the core of high vitality, from which the vitality intensity decreased towards the surroundings. The vitality of 26 public spaces (except space 14) was the lowest in winter, which was also in line with the characteristics of winter being the low season for tourism.

### 3.2. Spatial Autocorrelation Analysis

Global Moran’s I statistics were used to calculate the spatial autocorrelation of variables in rural areas in Jiangning District. The tests in Table 2 indicate that the Moran’s Index values for the 26 optional variables were greater than zero and that the P-statistic values for other independent variables than interference immunity, plant community structure, green looking ratio, seasonal change, plant growth potential, and topographic richness were smaller than 0.05 (the significance level was set to 0.05 here). Hence, the null hypothesis was rejected. The results suggested that all the alternative, independent variables chosen for the present study had certain spatial autocorrelation. Besides, except for the above 6 variables, the observed values of other independent variables Z were positive and greater than 2.58, which further indicated that the alternative, independent variables had strong spatial aggregation characteristics, which met the conditions for building models.

### 3.3. Selection of Explanatory Variables

(1) Tourists

As shown from Model 1 in Table 3, the VIF values of several variables, such as walking distance, space type and interference immunity, were greater than 7.5, indicating that there was an obvious multi-collinearity problem. Seven explanatory variables affecting tourist vitality in public spaces were obtained through stepwise linear regressions: they were accessibility, plant species richness, color composition, green looking ratio, water area, suburban, and recreational facilities. The probability of all variables was less than 0.05, and other independent variables were related to the space vitality of tourists. Besides, the VIF value of each variable calculated by Model 2 was smaller than 10, which was interpreted as there being serious multi-collinearity among the independent variables chosen for Model 2. In order to ascertain if the explanatory variable in the model has a consistent association with the dependent variable, Koenker’s studentized Bruesch-Pagan (Koenker (BP)) statistics are utilized [38]. The tested model is steady state, which is the test’s null hypothesis. A p-value of less than 0.05 for a 95% confidence interval denotes statistically significant heteroscedasticity and/or unsteadiness in the model. Koenker’s (BP) value is 18.752 (*p* = 0.01), which shows that the statistical data is statistically significant and the model is non-stationary.

(2) Residents

As shown in Table 4, the stepwise linear regression resulted in 5 explanatory variables that affect the vitality of residents in public spaces, i.e., space type, accessibility, fitness facilities, shelter facilities, and seats. The probability values of all explanatory variables were smaller than 0.05, while other independent variables were significantly correlated with the vitality of residential space. Furthermore, Model 2 presented the VIF value for each variable, which was always less than 10, suggesting that there was no serious multi-collinearity among the explanatory variables chosen for Model 2. In addition, Koenker’s (BP) value is 14.182 (*p* = 0.03), which shows that the statistical data is statistically significant and the model is non-stationary, too.

#### The Relationship between Space Vitality and Environmental Factors

(1) Tourists

The comparison of results between the GWR model and multivariable linear regression model indicated that the geographically weighted regression model had better explanatory capacity, thus better suiting the analysis of data law. As shown in Table 5, the goodness of fit of the GWR model was 0.827, and the adjusted goodness of fit was 0.723, which was higher than that of the OLS model. Moreover, the corrected Akaike information criterion (AICc) value decreased from 306.582 to 200.809. When compared with the OLS value, the GWR result was smaller, and the difference was greater than three. Hence, the information provided by GWR was more specific and reliable than that by OLS.

**Figure 6:**
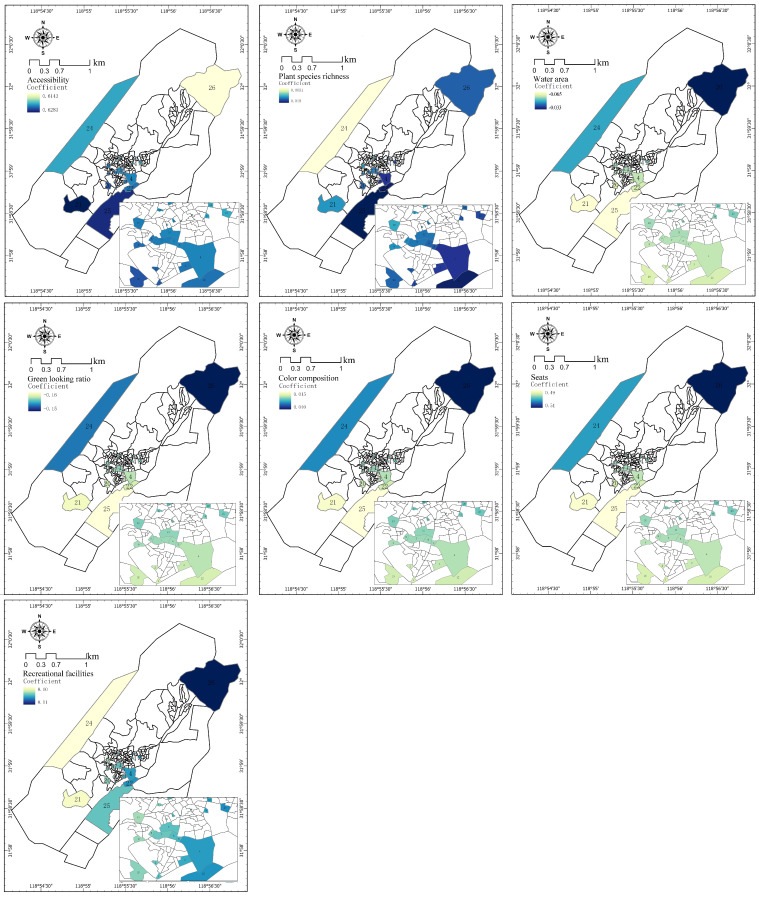
Spatial Patterns of Regression Coefficients between Tourist Vitality and the Factors of Environment.

The visualization of the tourist GWR model shows the spatial differentiation characteristics of the influence intensity of explanatory variables on tourist vitality in public spaces (Figure 6). The overall impact of accessibility, plant species richness, color composition, seats, and recreational facilities on the vitality of tourists in public spaces was positively correlated, while the green looking ratio and the water area were negatively correlated with the vitality of tourists in public spaces. Through the comprehensive regression coefficients of GWR model factors, the degree of impact on residents was in the following order:

Accessibility (0.623) > seats (0.494) > green looking ratio (−0.154) > recreational facilities (0.105) > water area (−0.042) > color composition (0.023) > species richness (0.015).

The GWR local coefficient visualization indicated that there were spatial differences in the impact of the space environment on tourist vitality. Among explanatory variables, accessibility of space had the most pronounced impact on tourists. The areas having the greatest impact on tourist vitality in public spaces were the public spaces, e.g., space 25 and space 21, on the central axis of the study area. This was due to the fact that the spaces were located on either side of the only main road in the rural community. The positive impact on the public spaces (e.g., space 26) in the northeast of the study area was the lowest. It could be understood that the higher the accessibility of public space, the higher the vitality of tourists in public space. The lower the accessibility, the lower the vitality of tourists in public spaces. Seats, recreational facilities, color composition and species richness, had a positive impact on tourists. Seats, recreational facilities and color composition had similar patterns of influence in space. These three explanatory variables were greatly affected by artificial construction during rural revitalization and development. It should particularly be noted that green looking ratio and water area were negatively related to tourist vitality, which suggests that tourist vitality in public space decreased with the increase in green looking ratio and water area. That is because it was often difficult for tourists to reach areas with excessively high green looking ratios and excessively large water areas. Moreover, since such public spaces were not planned properly, the regression coefficient was negative.

(2) Residents

The comparison of resident vitality results between the GWR and OLS models revealed found that the GWR model was better than the OLS model and could interpret the data better. Table 6 shows the final parameters in the GWR model. The goodness of fit of the model was 0.89, and the adjusted goodness of fit was 0.84, which was higher than that of the OLS model. Moreover, the AICc value decreased from 321.665 to 227.9. When compared with the OLS value, the GWR value was smaller and the difference was greater than 3. Hence, the information provided by GWR was more specific and reliable than that by OLS.

**Figure 7:**
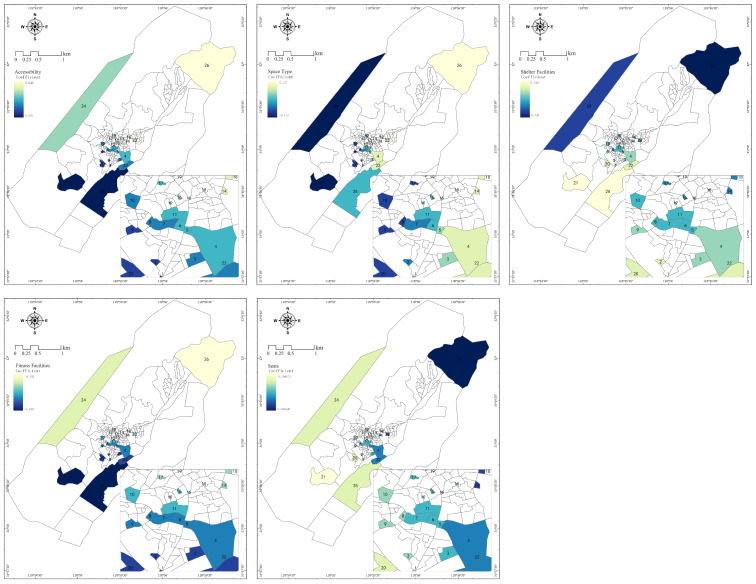
Spatial Patterns of Regression Coefficients between Resident Vitality and the Factors of Environment.

According to the analysis results of the OLS model and GWR model, the explanatory variables from plant landscape and topography and geomorphology had no immediate effect on resident vitality (Table 6 and Figure 7). However, there were more explanatory variables with significant effects from the perspective of space form and supporting facilities. The overall impact of accessibility, shelter facilities and seats on resident vitality in public spaces was positively correlated, while the space type and fitness facilities were negatively correlated. Obvious spatial heterogeneity was observed in all cases. Through the comprehensive regression coefficients of GWR model factors, the degree of impact on residents was in the following order:

Shelter facilities (0.342) > seats (0.296) > accessibility (0.063) > space type (0.113) > fitness facilities (−0.023)

It can thus be seen that the explanatory variable with the most significant impact on residents was shelter facilities, followed by seats. The shelter facilities and seats had a positive impact on resident vitality in the public spaces of Shecun. That is, the more shelter facilities and seats, the higher the resident vitality, and vice versa. As shown in the figure, the spatial distribution of the impact coefficients of the two explanatory variables was similar to space 21, and space 25 had the highest regression coefficient because they had minimal shelter facilities and seating, according to the spatial distribution of accessibility regression to each other. The impact coefficient and the influence intensity exhibited an increasing trend from southwest to northeast. Space 26 had the highest impact coefficient, probably because it was far away from settlements in a rural community since residents preferred to carry out activities in spaces near settlements, and the space had poor accessibility. The regression coefficient was maximum, and the central area had high spatial accessibility and high resident vitality, so the regression coefficient presented a positive correlation. Fitness facilities had a negative effect on resident vitality, which was consistent with the OLS result.

## 4. Discussion

### 4.1. How Do the Public Spaces in Suburban Rural Communities Affect Users?

In existing studies, population, economy and society are often considered the main components of rural community vitality. However, it is more controversial whether environmental factors should be directly related to the vitality of rural communities. Whereas some studies on the components of rural community vitality do not include environmental factors [39], some studies used the ecological environment as one of the vitality assessment factors. This study focuses on public spaces in suburban rural communities. In fact, it is the users of public spaces in suburban areas that are the source of vitality, but most studies focus on one of the two parties, tourists and residents. Therefore, the complex relationship between various groups and the public space environment can be understood by identifying the factors affecting the space vitality for tourists and residents. We agree that this may be a reasonable approach to the sustainable development of suburban rural communities.

Well then, how does the environment of public spaces in suburban rural communities affect various groups of people? This study is in line with the previous study. That is, various groups would directly or indirectly accept the impact on spatial perception and demand, thereby affecting space vitality [14]. Tourists were different from residents in terms of attributes and needs, as well as the perception of space in the same environment. Tourists were more inclined to choose spaces with cultural characteristics, convenient transportation and high suitability for relaxation. Since high-frequency locations of residents’ activities were more concentrated, they had lower demand for spaces when compared with tourists. An unexpected result was that water area was negatively correlated for both tourists and residents according to the OLS model results. The results of the tourist GWR model also showed that the water area had a negative impact even at the local scale. In other words, the larger the water area in the public space, the smaller the number of tourists and residents. This study is contrary to the previous study. The reason is that previous studies of space vitality were mostly focused on cities [31,40] but not integrated with a water area. On the one hand, the improvement of village infrastructures, such as the complete supply of piped water, has greatly reduced the dependence of villagers on rivers, so activities such as water collection and washing along rivers have gradually decreased. On the other hand, excessively large waters in rural communities are difficult for tourists to reach and conduct behavioral activities. Moreover, large areas of water in most rural communities are in a natural and pristine state, and they are not planned and designed, so they fall short of people’s needs for water proximity, making it impossible for people to stay and amuse themselves.

According to the result of the GWR model, road accessibility and seats had a significant positive impact on tourists and residents. The accessibility of rural public spaces not only affects space vitality but is also a dilemma faced by rural communities in China [41]. Studies have proved that, where walking was adopted, all people except the elderly and children could generally accept a distance of 400–500 m [42]. Therefore, they would choose the most natural route, avoid detours and obstacles, stairs and steps, and always select a straight route. Public service facilities such as seats and fitness facilities in the spaces played an important catalytic role in inducing user behavior. They promoted users’ certain behaviors and attracted more stays, thereby enhancing space vitality [43]. Studies have shown that seats was particularly important because residents and tourists attached top importance to physiological needs in outdoor public spaces [44]. However, since fitness facilities were arranged in closed and remote public spaces, they had a negative impact on residents, which is to say, fitness facilities in the space did not improve the space’s vitality. Some environmental factors had different effects on residents and tourists. For instance, the plant species richness and color composition had an impact on tourists but no impact on residents. Previous studies have proved that species richness and color composition of plants could significantly affect space vitality [45], but they did not distinguish between people. Public spaces with seasonal plants are the best ones [46]. The present study demonstrated that the seasonality of plants could affect the seasonal vitality distribution of tourists in public spaces, which was associated with the behavior patterns of different groups. For another example, space form had a positive impact on residents and had no impact on tourists because of the gathering psychology of local rural residents, “It is a lot of fun for people to watch others” [47].

### 4.2. Vitality Optimization Strategy for Public Spaces in Suburban Rural Communities

Public space vitality in rural communities of Shecun varied significantly, and the vitality of various groups was excessively concentrated, which brought about a waste of resources in most public spaces. The significance of public space is to gather people of different ages, populations and hobbies together so that all kinds of people with different life backgrounds gather in the spaces for communication, thus forming more extensive and complex social relations [48,49]. Tourists and residents are principal parts of suburban rural communities, and their behavior activities are carriers of public space vitality. Therefore, the presentation of optimization strategies depending on the needs of tourists and residents needs helps to further distinguish the space-time use of residents and tourists, enhance and enrich the experience of various user groups, promote positive and mutually beneficial on-site interaction between tourists and residents, thereby optimizing the public space vitality.

For tourists, it is advisable to enhance the continuity and integrity of road networks to improve accessibility, plan road networks as community groups, and organize communication through a “slow-traffic system” to integrate fast traffic and slow traffic, separate motor vehicles from the flow of people as appropriately as possible to reduce the impact of motor vehicle traffic on the lives of villagers. Field investigation indicated that tourists were cycling in groups, so a cycling system was to be established. Secondly, the color composition could be enhanced by increasing the number of varieties of plants. Priority is given to low shrubs, cover plants and tall trees so that the whole space is open with an airy line of sight. Thirdly, ribbons of water are used to link up the spaces, thereby attracting tourists to the dotted waters (ponds) in the spaces. This helps guide and gather tourists to areas close to water. The differences in water areas are used to optimize the vitality of public spaces. For instance, children from urban areas are less exposed to nature, so they are interested in recreational activities such as fishing and crayfish catching in the countryside. Therefore, fishing can be organized on small waters for children. Finally, the sites of recreational facilities are rationally arranged with reference to the spatial patterns of rural communities. Signage systems are set up to enhance the visibility of recreational facilities. Field investigation revealed that most of the tourists traveled in families in suburban rural communities. Recreational facilities in the Shecun Community were large entertainment items, and there were no recreational facilities specific to groups of people, especially children. Entertainment is the principal means of promoting children’s cognition [50].

Since many of the residents in the suburban rural communities were elderly people and children, the service facilities in public spaces had a great impact. The fitness facilities, shelter facilities and seats were rationally arranged, and the presence of related service facilities was enhanced to address residents’ habits in behavioral activities. Studies have proved that the residents in suburban rural communities had “herd behavior”, which is to say, they liked to gather in crowds. Hence, related service facilities were arranged in open or semi-open spaces for crowd activities. Since most of the elderly people in suburban rural communities took care of children for their sons/daughters, it was advisable to arrange rest and communication spaces for the elderly around the amusement facilities for children or to set up children’s fitness facilities in fitness venues. It is noteworthy that all shelter facilities in the public spaces of the study cases did not include barrier-free facilities despite the fact that most residents were elderly in suburban rural communities. Hence, it is necessary to reasonably improve the construction of barrier-free facilities and advisable to arrange safety facilities such as handrails where necessary to facilitate the occurrence of physical activity in the elderly, thereby minimizing the inconvenience and probability of accidents in the elderly.

### 4.3. Limitations and Insights for Future Research

There are still limitations and uncertainties in this study. First of all, although GPS data can be used as a representation of travelers’ activities, there is no software to process trajectories automatically, and this task is difficult and time-consuming. Future attempts could be made to combine cell phone signaling data with traditional surveys to extract more representative and complete information on the space vitality of rural communities. Secondly, the environmental indicators selected in this study were determined based on field surveys and relevant literature, but the level of economic development varies greatly among China and is not applicable to all regions. Therefore, the method can only be used as a reference for other provinces and cities. Finally, this study focuses on the built environment variables of suburban rural communities, while other variables (e.g., historical and cultural, socioeconomic, and crowd perceptions) can also have influences on space vitality. For example, users’ emotional perceptions were explored through Weibo comment data. It is one of the most popular social media platforms in China, and studies have been conducted to demonstrate the comprehensiveness of doctoral data [51]. Research could further explore the relationship between suburban rural community vitality and other variables to provide more reference for the planning design of suburban rural communities.

## 5. Conclusions

The rapid development of urbanization has led to the formation of a complex and varied area in suburban rural communities. It should particularly be noted that, with the intervention of rural tourism, the imbalance in the needs of different groups of people and landscape configuration in public spaces in suburban rural communities brought about the vitality imbalance in public spaces of suburban rural communities. However, the real needs of tourists and residents are ignored in the current planning of suburban rural communities in China, and more research focuses on tourists. Accordingly, the study proposes a method to reveal the characteristics of space vitality based on tourists and residents by using GPS data and cognitive map data for long-term tracking. In addition, OLS and GWR were used to probe into leading factors affecting the vitality of tourists and residents in four aspects, i.e., space form, plant landscape, topography and geomorphology, and related facilities. The balance of space vitality was maximized by comparing residents’ and tourists’ demand for spaces to propose differentiated strategies for balancing the vitality of public space.

Findings of the present study: (1) There were obvious seasonal changes and spatial distribution differences in the space vitality of tourists, while residents were concentrated in fixed public spaces regardless of season and time. (2) For tourists, the public space vitality in suburban rural communities was affected by seven factors, including accessibility, seats, green looking ratio, recreational facilities, water area, plant species richness, and plant color composition. The accessibility, plant species richness, color composition, seats, and recreational facilities positively affected the tourist vitality in public space, while green looking ratio and water area negatively affected the tourist vitality in public space. For residents, five factors, including shelter facilities, seats, accessibility, space type, fitness facilities, had significant effects on the public space vitality in suburban rural communities. The accessibility, space type, seats, and shelter facilities positively affected the resident vitality in public spaces, and only fitness facilities had negative effects. Such results were of great importance since they can be used by administrators and planners to make improvements. The information on public spaces attracting the most tourists and residents may help formulate and adjust the space configuration, facilities and services. Such results may also determine the actions to reduce the negative effects on various groups in support of more sustainable tourism in cities and rural revitalization. Therefore, it is vital that strategies for optimizing the public space vitality in suburban rural communities are established.

The contribution of this study is to propose a method to evaluate the public space vitality in suburban rural communities from the perspective of tourists and residents and to explore the environmental factors affecting the space vitality through GWR and OLS. Studying a large number of insights can inform policymakers and planners for future work, which has important implications for the vibration-oriented planning of suburban rural communities.

## Figures and Tables

**Table 1:** Environmental Factors and Description.

Categories	Environmental Factors	Content Description
Space form	Site area	Total site area (square meters)
	Green space area	Total green space area (square meters)
	Walking distance	Length of footpath
	Space type	Open (1), semi-open (2), closed (3)
	Accessibility	Spatial global integration value
	Interference immunity	Is there any ability to isolate from outside traffic and noise? Yes (1)/No (2)
Plant	Plant species richness	Number of species in plant community
	Plant community structure	Types of plant community structure, including arbor-shrub-grass, arbor-grass, and arbor-shrub, etc.
	Plant canopy density	Ratio of the total projected area of tree crown on the ground in sunlight to the total site area
	Percentage of indigenous plant	Varieties of indigenous plants/varieties of all plants
	Plant growth potential	Plant growth status. Good (3)\Medium (2)\Poor (1)
	Green looking ratio	Green proportion
	Seasonal change	Richness of seasonal changes of plant communities, including “no change throughout the four seasons”, “change in one season”, and “change in two seasons”, etc.
	Color composition	Richness of color composition of plant communities, including “green only” and “multiple colors”, etc.
Topography and geomorphology	Water area	Total water area (square meters)
	Topographic richness	Is there any terrain? Yes (1)/No (2)
Supporting facilities	Seats	Number/length of seats
	Shelter facilities	Quantity of shelter facilities (pavilions, corridors, and flower stands, etc.)
	Sanitation facilities	Quantity of sanitation facilities (restrooms and trash cans)
	Fitness facilities	Quantity of established fitness equipment
	Recreational facilities	Children’s amusement facilities, recreational facilities for the elderly (chess and cards rooms, senior citizens activity centers, etc.), and entertainment items

**Table 2:** Moran’s Index for Environmental Factors.

Level-One Variables	Level-Two Variables	Moran I	*p*	z
Space form	Site area	0.02	0.01	2.61
	Walking distance	0.04	0.01	2.87
	Green space area	0.004	0.00	2.55
	Space type	0.03	0.01	2.77
	Accessibility	0.06	0.00	3.08
	Interference immunity	0.025	0.01	2.63
Plant landscape	Plant species richness	0.09	0.01	2.61
	Plant community structure	0.21	0.00	0.18
	Plant canopy density	0.07	0.03	2.64
	Percentage of indigenous plant	0.04	0.01	2.59
	Plant growth potential	0.02	0.66	0.43
	Green looking ratio	0.04	0.88	0.14
	Seasonal change	0.06	0.01	0.17
	Color composition	0.04	0.00	2.72
Topography and geomorphology	Water area	0.04	0.00	2.59
	Topographic richness	0.08	0.20	1.25
Supporting facilities	Seats	0.05	0.00	3.15
	Shelter facilities	0.23	0.04	2.69
	Fitness facilities	0.04	0.00	2.86
	Recreational facilities	0.17	0.01	2.67
	Sanitation facilities	0.05	0.00	2.56

**Table 3:** Results of the Linear Regression Analysis-Tourists. * *p* < 0.05.

Model 1	**Variables**	**Coefficients**	**Standard Deviations**	**T Values**	***p* Values**	**VIF**
	Walking distance	0.650	0.273	2.382	0.096	415.495
	Space type	6.086	40.909	0.149	0.886	6.048
	Accessibility	1945.690	627.056	3.103	0.004 *	22.995
	Interference immunity	142.467	88.672	1.607	0.201	17.237
	Plant species richness	−2.949	8.081	−0.365	0.734	27.883
	Percentage of indigenous plant	−323.309	228.943	−1.412	0.247	32.632
	Plant growth potential	57.741	26.875	2.148	0.118	7.725
	Green looking ratio	81.117	268.371	0.302	0.776	7.698
	Seasonal change	−77.335	38.716	−1.998	0.136	10.376
	Color composition	12.440	45.243	0.275	0.795	14.822
	Water area	−0.006	0.002	−2.512	0.086	394.628
	Topographic richness	6.788	44.645	0.152	0.884	4.476
	Seats	9.405	3.138	2.997	0.056	18.994
	Shelter facilities	−65.975	19.257	−3.426	0.035 *	4.671
	Sanitation facilities	45.052	38.006	1.185	0.315	12.283
	Fitness facilities	33.438	25.292	1.322	0.272	11.058
	AICc	414.367				
Model 2	Accessibility	1118.597	174.388	6.414	0.000 *	1.650
	Plant species richness	9.301	2.741	3.394	0.000 *	2.976
	Green looking ratio	−209.119	206.676	−2.01	0.003 *	1.416
	Color composition	96.764	22.624	4.277	0.000 *	3.288
	Water area	−0.065	0.018	−2.513	0.000 *	2.015
	Seats	7.527	1.209	6.221	0.000 *	2.620
	Recreational facilities	−40.876	19.439	−2.102	0.009 *	2.726
	R^2^	0.81				
	Adj R^2^	0.709				
	F	5.90				
	Sig.	0.000				
	AICc	306.582				

**Table 4:** Results of the Linear Regression Analysis—Residents. * *p* < 0.05.

Model 1	**Variables**	**Coefficients**	**Standard Deviations**	**T Values**	***p* Values**	**VIF**
	Walking distance	−0.439	0.336	−1.308	0.276	610.101
	Space type	−68.940	41.467	−1.663	0.189	6.023
	Accessibility	308.755	678.061	0.455	0.674	26.058
	Interference immunity	31.759	88.697	0.358	0.738	16.715
	Plant species richness	−11.069	8.937	−1.239	0.298	33.046
	Percentage of indigenous plant	−127.381	259.742	−0.490	0.652	40.706
	Plant growth potential	12.870	28.444	0.452	0.676	8.386
	Green looking ratio	353.722	251.460	1.407	0.248	6.550
	Seasonal change	−121.472	39.133	−3.104	0.051	10.274
	Color composition	20.492	45.069	0.455	0.675	14.254
	Water area	−0.005	0.004	−1.189	0.314	984.872
	Topographic richness	−78.619	44.950	−1.749	0.173	4.397
	Seats	0.260	3.446	0.075	0.942	22.199
	Shelter facilities	42.491	17.705	2.400	0.095	3.826
	Sanitation facilities	−62.730	51.623	−1.215	0.305	21.962
	Fitness facilities	−20.736	28.623	−0.724	0.516	13.724
	AICc	497.971				
Model 2	Space type	−55.387	33.174	−1.669	0.012 *	2.285
	Accessibility	834.550	308.415	2.705	0.002 *	3.195
	Shelter facilities	82.314	82.314	5.393	0.000 *	1.685
	Fitness facilities	−16.211	12.290	12.290	0.004 *	1.500
	Seats	2.227	1.160	1.919	0.014 *	1.492
	R^2^	0.829				
	Adj R^2^	0.73				
	F	6.987				
	Sig.	0.000				
	AICc	321.665				

**Table 5:** Calculation Results of GWR Model—Tourists.

R^2^	Adj R^2^	AICc	Sigma-Squared MLE
0.827	0.723	200.809	0.042

**Table 6:** Calculation Results of GWR Model—Residents.

R^2^	Adj R^2^	AICc	Sigma-Squared MLE
0.89	0.84	227.9	0.106

## Data Availability

Not applicable.
